# Novel Probiotic Lactic Acid Bacteria Were Identified from Healthy Infant Feces and Exhibited Anti-Inflammatory Capacities

**DOI:** 10.3390/antiox11071246

**Published:** 2022-06-24

**Authors:** Binbin Li, Li-Long Pan, Jia Sun

**Affiliations:** 1 State Key Laboratory of Food Science and Technology, Jiangnan University, Wuxi 214122, China; 7200112012@stu.jiangnan.edu.cn; 2 School of Food Science and Technology, Jiangnan University, Wuxi 214122, China; 3 School of Medicine, Jiangnan University, Wuxi 214122, China

**Keywords:** probiotic, lactic acid bacteria, isolation, infant feces, anti-inflammation, antioxidants

## Abstract

The current study aims to evaluate the probiotic potential of lactic acid bacteria isolated from infant feces, and select candidates to be used as potential antioxidants for the treatment of oxidative stress-related diseases; To meet the criteria for probiotic attributes, the isolates were subjected to various in vitro tests and 16S rRNA genotypic characterization. Besides, anti-inflammatory and anti-oxidative effects of selected isolates were separately assessed by real-time quantitative PCR and Western blot; The selected strains belonged to *Lactobacillus gasseri*, *Lactiplantibacillus plantarum* and *Lacticaseibacillus rhamnosus*. Notably, three selected strains (*L. gasseri* FWJL-4, *L. plantarum* Fjias-5 and *L. rhamnosus* FSJ-13) particularly *L. gasseri* FWJL-4 significantly down-regulated mRNA expression levels of tumor necrosis factor α (TNFα), Interleukin-6 (IL-6) and IL-1β. Most importantly, three strains-treated RAW 264.7 murine macrophages displayed enhanced activities of antioxidant enzymes and reduced H_2_O_2_ production, which were associated with the enhanced expression levels of nuclear factor-erythroid 2 related factor 2 and heme oxygenase-1; Three selected strains, particularly *L. gasseri* FWJL-4, are good candidates that merit additional in vivo investigation for the validation and application of their health-promoting effects.

## 1. Introduction

Probiotics are live microorganisms that when consumed in adequate amounts, can exert beneficial effects on the host [1]. Well-known probiotic microorganisms include genera of lactic acid bacteria (LAB) (*Lactococcus*, *Lactobacillus*, *Streptococcus*, *Pediococcus*, *Leuconostoc* and *Enterococcus*), *Bifidobacterium*, *Bacillus*, as well as yeasts [1,2,3,4]. Among them, the genera *Lactobacillus*, *Limosilactobacillus*, *Lacticaseibacillus*, *Lactiplantibacillus*, etc. are the earliest found and most studied probiotics, which are Gram-positive and catalase-negative probiotics and have been shown to provide health benefits to the human host. As reported, LAB are natural inhabitants of the human gastrointestinal tract and play a critical role in restoring the human gut microbiota homeostasis [5]. In addition, LAB exhibit positive effects on various aspects of human health including nutrition, metabolism, immunity, and defense against pathogens [6]. *Lacticaseibacillus casei*, *Lacticaseibacillus paracasei*, *Lactobacillus acidophilus*, *Limosilactobacillus fermentum*, *Lactiplantibacillus plantarum*, *Limosilactobacillus reuteri*, *Lacticaseibacillus rhamnosus*, *Lactobacillus gasseri* and *Lactobacillus crispatus* are common probiotics existing in animal and human gastrointestinal and digestive systems [7]. These strains have been isolated from different environmental sources such as fermented foods [8] and human samples [9].

As reported, LAB can produce a high amount of lactic acid and other metabolites such as bacteriocins [10], short-chain fatty acids [11], soluble mediators [12], and exopolysaccharides [13]. Consequently, they possess the ability to withstand harsh conditions in the human body (such as salivary enzymes, low pH, and intestinal juice), colonize gut epithelial cells, and inhibit pathogens and reactive oxygen species (ROS) related to gut diseases, thus helping to maintain gut microbiota balance and immune homeostasis and exert physiological roles in human health [14,15]. 

Oxidative stress is an excessive production of ROS, which can provoke the onset and progression of various diseases, including diabetes [16], cancer [17] and cardiovascular diseases [18], etc. Accumulating studies have suggested that intestinal flora metabolites may participate in the modulation of inflammation-related diseases through regulating oxidative stress [19,20,21]. Despite the evident potential, knowledge is scarce about the antioxidant effects of probiotics, as an alternative for various disease protection against oxidative damage. The detailed mechanisms of probiotics in mediating inflammation and oxidative stress-related diseases remain to be fully explored. Currently, despite great efforts in the identification of probiotic strains, their functional substances (either metabolites or components) haven’t been specified.

The current study aims to isolate and identify novel bacterial strains belonging to the LAB from healthy infants, which can be used as potential probiotics against inflammation and oxidative stress-related human diseases. The candidate probiotic strains or their metabolites can be potentially used as novel antioxidants. 

## 2. Materials and Methods

### 2.1. Sample Collection and Strain Isolation

A total of 100 stool samples of healthy infants (63 boys and 37 girls; without gastrointestinal tract diseases, allergic diseases and congenital diseases) aged under 3 weeks, were collected for the study. The present study was approved by the ethics committee of the Affiliated Wuxi Children’s Hospital of Nanjing Medical University [WXCH2018-08-007]. Written informed consent was obtained from the parents after a careful explanation of the research. After collection, samples were transferred to de Man-Rogosa-Sharpe (MRS) broth (purchased from Hope Bio-Technology Co., Qingdao, China) and cultured at 37 °C for 48–72 h. The MRS broth was composed of 2 g/L of dipotassium hydrogen phosphate, 20 g/L glucose, 0.2 g/L magnesium sulfate heptahydrate, 0.05 g/L manganous sulfate tetrahydrate, 8 g/L beef extract, 10 g/L peptone, 5 g/L sodium acetate trihydrate, 2 g/L triammonium citrate and 4 g/L yeast extract. After that, the samples were diluted with sterile saline and spread onto MRS agar medium, and incubated for 48–72 h at 37 °C under anaerobic conditions in an anaerobic workstation (Electrotek, West Yorkshire, UK). LAB colonies were purified twice using MRS agar medium. Then, the obtained bacterial isolates were used for morphological and biochemical characterization. The reference strain *L. rhamnosus* GG (LGG, ATCC 53103), gut-related pathogens, including *Staphylococcus aureus* ATCC 25923, *Salmonella enterica* ATCC 14028, *Escherichia coli* ATCC 25922, *Listeria monocytogenes* ATCC 13932 were obtained from the American Type Culture Collection (ATCC). *Enterococcus faecalis* E27 was obtained from the Culture Collection Center of Jiangnan University (Wuxi, China). Selected strains were kept in-house culture collection of the School of Food Science and Technology, Jiangnan University at −80 °C in 30% glycerol (*v*/*v*). 

### 2.2. Morphological and Biochemical Characterization

The Gram staining test was used to characterize bacterial morphological properties. Biochemical properties were evaluated using the catalase test and analysis of carbohydrate fermentation. The biochemical identification tube system was purchased from Hope Bio-Technology Co., to analyze carbohydrate fermentation profiles of bacterial isolates. Fifteen kinds of carbohydrates were selected including glucose, galactose, maltose, mannose, lactose, sucrose, inositol, sorbitol, L-xylose, fructose, L-arabinose, rhamnose, cellobiose, esculin and mannitol. Each tube contained a specific carbohydrate. The signal bacterial colony was incubated in the relevant tube and cultured at 37 °C for 48–72 h under anaerobic conditions. The positive results were recorded according to the manufacturer’s instructions. The growth abilities of strains in the presence of 3% and 4.5% NaCl, and at 15 °C and 45 °C were also assessed [22,23]. Finally, catalase-negative and Gram-positive strains were selected as potential probiotic strains. The selected bacterial isolates were stored in 30% glycerol (*v*/*v*) at −80 °C for further use.

### 2.3. Preliminary Selection of LAB Using Specific Primers

The Gram-positive and catalase-negative strains were selected and used for further analysis. Bacterial genomic DNA of all strains was extracted using a TIANamp Bacteria DNA Kit (Tiangen Biotech Co., Beijing, China) according to the manufacturer’s instructions. Specific primers of LAB were used (forward: 5′-GCYGGTGCWAACCCNGTTGG -3′; reverse: 5′-AANGTNCCVCGVATCTTGTT-3′) [24]. The PCR mix contained 25 µL of 12.5 µL of 2 × Taq Plus Master Mix (CWBIO Biotech Co., Beijing, China), 1 µL of each primer (10 μmol/L), 1 µL of DNA templates, and 9.5 µL of ddH_2_O. The amplification program was performed over 30 cycles of 94 °C for 30 s, 58 °C for 30 s and 72 °C for 2 min, with a final extension step of 10 min at 72 °C. A 1% agarose gel electrophoresis was performed to determine the sizes and amounts of the amplicons. The positive strains were then selected for further analysis.

### 2.4. Antimicrobial Activity Assessment

To detect the inhibitory effects of selected LAB on several major gut-related pathogens, including *Staphylococcus aureus* ATCC 25923 (LB medium), *Salmonella enterica* ATCC 14028 (BHI medium), *Escherichia coli* ATCC 25922 (LB medium), *Listeria monocytogenes* ATCC 13932 (BHI medium), *Enterococcus faecalis* E27 (MRS medium), the Oxford cup method was used. In detail, the bacterial isolates cultured in MRS broth at 37 °C for 24–48 h were centrifuged at 12,000 rpm for 10 min. Then the supernatants were collected. The pathogens were incubated at 37 °C for 16–24 h (approximately 1 × 10^6^ CFUs/mL) and spread onto a separated MRS medium. The sterilized Oxford cup was carefully placed and pressed onto the medium, and 200 μL of bacterial supernatants were distributed into the cups. After incubation at 37 °C for 24 h, the antimicrobial activity of the strains was recorded as a growth-free inhibition zone around the Oxford cups. The diameter of the inhibition zones was scored as follows: less and equal to 7 mm (negative, −); 7–15 mm (weak), >15 mm (strong). The antibiotic penicillin (30 mg/mL) was used as a positive control. A corresponding liquid medium was used as the negative control. Each test was conducted in triplicate.

### 2.5. Tolerance to Simulated Digestive Tract Conditions

The simulated digestive juice was prepared according to previous report with minor modifications [25]. Artificial saliva (pH 6.9) consisted of 3 g/L α-amylase (Sigma-Aldrich Co., St. Louis, MO, USA) in a sterile solution, which contained 6 g/L NaCl, 0.2 g/L CaCl_2_, 2 g/L KCl and 1 g/L NaHCO_3_. Simulated gastric fluid (pH 2) was prepared by dissolving 3.0 g/L pepsin from porcine gastric mucosa (Sigma-Aldrich) in a sterile solution, which contained 1.1 g/L KCl, 3 g/L NaCl, 0.6 g/L NaHCO_3_ and 0.15 g/L CaCl_2_. Artificial small intestinal juice (pH 7.4) was prepared by dissolving 3 g/L bile salt (Sigma-Aldrich), 0.1 g/L lipase (Sigma-Aldrich) and 1 g/L pancreatin (Sigma-Aldrich) in sterile solution, which consisted of 5 g/L NaCl, 0.6 g/L NaHCO_3_, 0.3 g/L CaCl_2_ and 0.6 g/L KCl. All the solution was filtered through a 0.22-µm filter before use. Selected bacterial cells (1 mL, 1 × 10^8^–10^9^ CFUs/mL) were firstly suspended in 1 mL artificial saliva for 5 min. The cells were then centrifuged (4 °C, 12,000 rpm, 2 min) and resuspended in 2 mL gastric fluid and incubated at 37 °C for 2 h. Subsequently, bacterial cells were recentrifuged (4 °C, 12,000 rpm, 2 min) and resuspended in 2 mL simulated small intestinal fluid and incubated at 37 °C for 2 h. Finally, the bacterial suspensions were diluted and plated on MRS agar and cultured at 37 °C for 36–48 h under anaerobic conditions. The total number of colonies was counted and the survival rates were recorded according to the formula as follows:

$$Survival\;rate\;\left(\%\right)=\frac{LogN1}{LogN0}\times 100$$

*N*1 represents the total count of strains after simulated digestive juice treatment. *N*0 represents the total count of strains before simulated digestive juice treatment. Each test was conducted in triplicate.

### 2.6. Adherence to Intestinal Epithelial Cells

The adhesion ability of the bacterial isolates to human intestinal epithelial cells was measured according to the procedures described by Balthazar et al. [26]. The human colon adenocarcinoma (Caco-2) cell line (obtained from China Cell Bank) was used as the target cell to examine cell adhesion of selected strains. Caco-2 cells were cultured in Dulbecco’s Modified Eagle’s Medium (DMEM) (Hyclone, Logan, UT, USA) supplemented with 1% penicillin-streptomycin (Ameresco, United States) and 10% fetal bovine serum (FBS) (Wisent, ST-BRUNO, QC, Canada) at 37 °C, 5% CO_2_. Firstly, Caco-2 cells were seeded in 24-well culture plates (2 × 10^5^ cells/well) and incubated until the confluency. Before adhesion, the medium in each well was washed with sterile PBS and replaced with a prewarmed fresh medium without antibiotics. Overnight cultured fresh bacterial cells (10^8^ CFUs/mL) were washed twice with sterile PBS and added to each well. Following co-incubation for 3 h at 37 °C, cells were washed twice. Then, the cells were treated with trypsin/EDTA (Sigma-Aldrich) at 37 °C for 3 min. The suspension from each well was transferred to serial saline for 10-fold dilution and plated onto MRS agar plates. After 36–48 h of incubation at 37 °C, the total number of colonies was counted under anaerobic conditions. The adhesion rate was calculated by measuring the total bacterial counts before and after bacteria adhered to the Caco-2 cells.

$$Adhesion\;rate\;\left(\%\right)=\frac{C1}{C0}\times 100$$

*C*1 represents the total bacterial counts adhered to Caco-2 cells. *C*0 represents the total bacterial counts of strains before treatment. Each test was conducted in triplicate. 

### 2.7. Antibiotic Resistance Assay

Eight antibiotics including four cell membrane/wall inhibitors (penicillin, ampicillin, polymyxin and vancomycin) and four protein synthesis inhibitors (streptomycin, erythromycin, chloramphenicol and kanamycin), are common antibiotics used for clinical infection [27]. The concerns regarding specific strains are long-term use may develop antibacterial resistance [28]. All antibiotics were purchased from Sangon Biotech Co., Shanghai, China. Each antibiotic was dissolved with a proper solution and filtered before use. 

Bacterial strains were incubated in MRS broth supplemented with different final concentrations (2, 4, 8, 16, 32, 64, 128, 256, 512 and 1024 μg/mL) of antibiotics for 24 h incubation at 37 °C, and assayed in triplicate in a microplate reader (OD at 610 nm) [29]. MICs (minimum inhibitory concentrations) were considered the lowest concentrations of antibiotics that could inhibit strain growth, which was used to evaluate the antibiotic resistance of selected strains.

### 2.8. Strain Identification Using 16S rRNA Sequence Analyses

Primers used for amplifying the complete sequence of 16S rRNA were 27F (5′-AGAGTTTGATCCTGGCCTCA-3′) and 1492R (5′-GGTTACCTTGTTACGACTT-3′) [30]. The PCR mix contains 25 µL of 12.5 µL of 2 × Taq Plus Master Mix, 1 µL of each primer (10 μ mol/L), 1 µL of DNA templates, and 9.5 µL of ddH_2_O. The amplification program was performed over 30 cycles of 94 °C for 30 s, 55 °C for 30 s and 72 °C for 2 min, with a final extension step of 10 min at 72 °C. A 2% agarose gel electrophoresis was performed to determine the sizes and amounts of the amplicons. The nucleotide sequences were used for sequence identity analysis (http://www.ncbi.nlm.nih.gov/blast, 16 S rRNA database, accessed on 27 May 2022). 

### 2.9. Cytokine Measurement 

Murine macrophages (RAW 264.7, obtained from China Cell Bank) were cultured in DMEM medium at 37 °C in a 5% CO_2_ humidified incubator [9]. A density of 1 × 10^5^ cells/mL was seeded in each well of a 6-well culture plate for 24 h. The culture supernatants of three different strains (OD_600_ of 1.0 units) (*L. gasseri* FWJL-4, *L. plantarum* Fjias-5 and *L. rhamnosus* FSJ-13) and positive control (LGG, OD_600_ of 1.0 units) were administrated to each well for 1 h, and then lipopolysaccharides (LPS, 1 μg/mL) were added for 20 h. The cell supernatants were obtained by centrifugation and filtration using a 0.22-μm membrane. According to the manufacturer’s protocol, Trizol reagent (Invitrogen Corp., Carlsbad, CA, USA) was used to exact total RNA from RAW 264.7 cells. The mRNA expression levels of interleukin-10 (IL-10), IL-6, IL-1β and tumor necrosis factor (TNFα) were determined using the CFX Connect Real-Time System (Bio-Rad, Hercules, CA, USA). The relative mRNA expression levels were normalized using the mRNA levels of β-actin. Primers used for real-time quantitative PCR (RT-qPCR) were indicated in Table 1.

### 2.10. Determination of Antioxidant Enzymes and H_2_O_2_ Production

RAW 264.7 cells (1 × 10^5^ cells/mL) were seeded in each well of a 24-well culture plate for 24 h. The culture supernatants of three different strains (OD_600_ of 1.0 units) (*L. gasseri* FWJL-4, *L. plantarum* Fjias-5 and *L. rhamnosus* FSJ-13) and positive control (LGG, OD_600_ of 1.0 units) were added to each well for 1 h, and then 1 μg/mL of LPS were added for 20 h. After that, the cells were collected for the determination of superoxide dismutase (SOD), glutathione peroxidase (GPx), glutathione (GSH) and oxidized glutathione (GSSG) using assay kits (Beyotime Biotechnology, Shanghai, China) according to the manufacturer’s instructions. For H_2_O_2_ measurement, the cells were firstly treated with culture supernatants of three different strains for 1 h, and then treated with 1 μg/mL of LPS for 2 h, 4 h, 6 h, 8 h, 10 h and 12 h, respectively. After LPS treatment, cells were washed and incubated with Amplex Red (50 μM, Beyotime) in the dark at 37 °C for 30 min [31]. Subsequently, cells were washed and the fluorescence density was measured at OD571 nm by a Varioskan LUX Multimode Reader (Thermo Fisher Scientific, Waltham, MA, USA). 

### 2.11. Western Blotting 

RAW 264.7 cells (1 × 10^5^ cells/mL) were seeded in each well of a 6-well culture plate for 24 h. The culture supernatants of three different strains (OD600 of 1.0 units) (*L. gasseri* FWJL-4, *L. plantarum* Fjias-5 and *L. rhamnosus* FSJ-13) were added to each well for 1 h, and then 1 μg/mL of LPS were added for 6 h. A Nuclear and Cytoplasmic Protein Extraction Kit (Beyotime) was used to extract the cell proteins. Equal amounts of protein were separated by electrophoresis (Mini-PROTEAN^®^ Tetra cell system, Bio-Rad) in 10% sodium dodecyl sulfate-polyacrylamide gel electrophoresis and then transferred to a nitrocellulose membrane (Millipore, Bedford, MA, USA). The membranes were firstly blocked with 5% nonfat milk and then incubated with respective primary antibodies containing 5% BSA overnight at 4 °C. The membranes were then incubated with horseradish peroxidase-conjugated anti-rabbit secondary antibodies (1:5000, Thermo Fisher Scientific) for 2 h. The blots were visualized by ChemiDoc Imager (Bio-Rad) after enhancing chemiluminescence reaction. Densitometric analyses of bands were quantified using Image Lab software (Version 3.0, Bio-Rad) with β-actin or histone H3 as the internal control. The following primary antibodies were used: polyclonal rabbit anti-Nrf-2 (Cat#16396-1-AP, 1:1000, Proteintech Group, Rosemont, IL, USA), polyclonal rabbit anti-HO-1 (Cat#10701-1-AP, 1:1000, Proteintech Group), monoclonal rabbit anti-histone 3 (Cat#9717, 1:1000, Cell Signaling Biotechnology, Danvers, MA, USA) and monoclonal rabbit anti-β-actin (Cat#AC026, 1:5000, Abclonal Biotechnology, Wuhan, China).

### 2.12. Statistical Analysis

Results were reported as mean ± SD of three triplicates. Differences among three or more groups were determined using one-way analysis of variance (ANOVA) followed by the Tukey *post hoc* test. Statistical significance was defined as * *p* < 0.05; ** *p* < 0.01; *** *p* < 0.001. Graphical presentations were generated by Graphpad Prism version 8.3.

### 2.13. Data Availability

Nucleotide sequence data reported are available in the Genbank database under the accession numbers: MZ220367 (FRY-2), MZ220368 (FXHB-6), MZ220369 (Fjias-5), MZ220370 (FRY-4X), MZ220371 (FWJL-4), MZ220372 (FZL-2), MZ220373 (FRY-6), MZ220374 (FSJ-13), MZ220375 (FZSJ-7), MZ220376 (FXHB-2), MZ220377 (FWJL-5), MZ220378 (FSJ-4X).

## 3. Results

### 3.1. Morphological and Biochemical Test Results

250 microbial strains were isolated from healthy infant feces. Among these strains, 27 isolates were selected based on morphological and biochemical tests. All strains were rod, Gram-positive and catalase-negative. They were able to grow in 3% and 4.5% NaCl (*w*/*v*), as well as at 45 °C. However, only eight strains could grow at the temperature of 15 °C. Carbohydrate fermentation profiles of each strain were recorded. The results showed that all strains were able to ferment glucose, galactose, maltose, mannose, lactose, sucrose, inositol, inositol, L-xylose, fructose, L-arabinose, rhamnose, cellobiose and esculin, and not able to ferment sorbitol and mannitol, preliminarily confirming that they belong to LAB. 

### 3.2. Specific Primers Amplification for LAB

**Figure 1:**
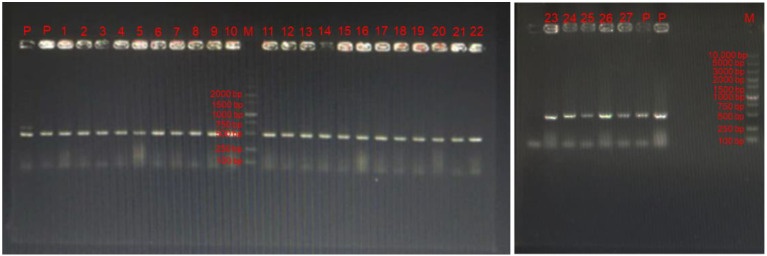
The specific-PCR detection of 27 bacterial isolates. “P” represents positive LAB (LGG). “M” represents the DNA marker. Numbers 1–27 are strains selected in the present study.

The selected strains were then used for PCR detection using specific primers of LAB [24]. Compare to reference strain LGG, the product length of 27 isolates was 500 bp, which was preliminarily considered as LAB (Figure 1).

### 3.3. Antimicrobial Test Results

Twenty-seven bacterial isolates were used to evaluate antimicrobial activities against five pathogens using their cell-free culture supernatants. As shown in Table 2, seven isolates (FMM-1, FLY-17, FMM-7, FHHY-7, FSJ-6, FLT6-11 and FQM-3X) showed either poor or no activity against five pathogens, while other bacterial isolates exhibited better antimicrobial activities. Compared to penicillin, Fjias-5, FWJL-4, FSJ-11, FSJ-13, FXHB-2, FSJ-4X, FXHB-6, FXHB-2X and FHHY-1 showed strong antimicrobial activities against more than three pathogens. Notably, 23 out of 27 isolates had a strong inhibitory effect on *Enterococcus faecalis* E27.

### 3.4. Tolerance to Simulated Digestive Tract Condition

**Figure 2:**
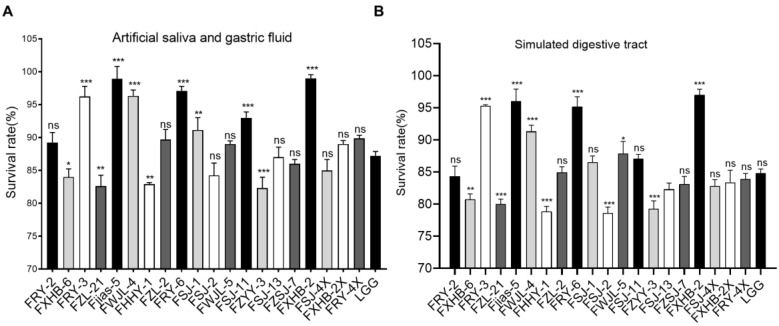
The survival rates of the selected strains in simulated digestive tract conditions. All data were expressed as mean ± SD (*n* = 3 independent experiments). Statistical significance was defined as * *p* < 0.05; ** *p* < 0.01; *** *p* < 0.001. “ns”: no significance. (**A**) Differences of selected strains in artificial salvia and gastric fluid compared with reference strain LGG. (**B**) Differences of selected strains in simulated digestive tract compared with LGG.

The bacterial isolates with significant antimicrobial activities (20 isolates) were selected to assess their survival rates in simulated digestive juice. As shown in Figure 2A,B, the artificial saliva and gastric fluid treatment (Figure 2A) had a less significant effect on the survival of bacteria than that of the simulated digestive juices (Figure 2B). However, the survival rates of most strains in the artificial saliva and gastric fluid were almost the same as those in the simulated digestive juice, suggesting that the pH of digestive juice played a crucial role. Compared with the reference strain LGG, several tested strains showed a higher level of tolerance (*p* < 0.05). In particular, five strains, namely FRY-3, Fjias-5, FWJL-4, FRY-6 and FXHB-2, exhibited >90% of survival rates in simulated digestive juice (Figure 2B). In contrast, most strains (FZL-21, FHHY-1, FSJ-2, FZYY-3, FSJ-13, FXHB-2X, FRY-4X) showed a lower level of tolerance than the reference strain LGG (*p* < 0.05) (Figure 2B). 

### 3.5. Adherence to Caco-2 Cells 

**Figure 3:**
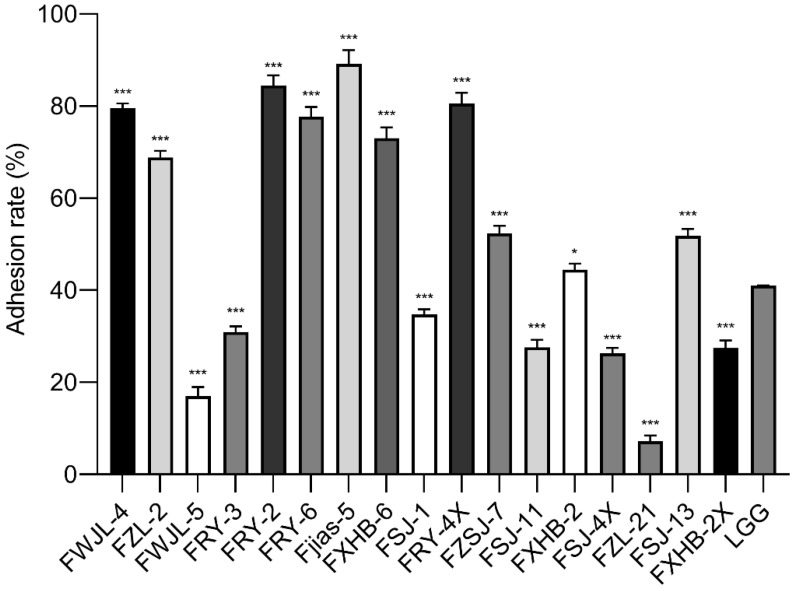
The adhesion ability of the selected strains to Caco-2 cells in vitro. All data were expressed as mean ± SD (*n* = 3 independent experiments). Statistical significance was defined as * *p* < 0.05; *** *p* < 0.001. Reported statistical significance refers to comparisons with LGG.

Based on the tolerance test results, three strains (FHHY-1, FSJ-2 and FZYY-3) with lower survival rates were excluded and the remaining strains were further tested for their adhesion ability to Caco-2 cells in vitro. As shown in Figure 3, ten bacterial isolates (FWJL-4, FZL-2, FRY-2, FRY-6, Fjias-5, FXHB-6, FRY-4X, FZSJ-7, FXHB-2 and FSJ-13) showed a higher adhesion ability than the reference strain LGG (*p* < 0.05). Among them, FRY-2, FRY-4X and Fjias-5 exhibited excellent adhesion rates (>80%). In contrast, other isolates, including FWJL-5, FRY-3, FSJ-1, FSJ-11, FSJ-4X, FZL-21 and FXHB-2X, showed a significantly lower adhesion ability (mostly < 30%) than LGG (*p* < 0.05). 

### 3.6. Antibiotic Resistance 

The MICs of tested strains in the presence of different concentrations of antibiotics were shown in Table 3. Most selected strains exhibited resistance to three antibiotics (kanamycin, vancomycin and polymyxin), especially to polymyxin, but were sensitive to other antibiotics. Nine strains including FRY-2, FXHB-6, FRY-3, FZL-21, Fjias-5, FRY-4X, FWJL-4, FZL-2 and FRY-6 showed antibiotic sensitivity to cell wall inhibitors such as penicillin G, ampicillin, and vancomycin. Furthermore, almost all strains were sensitive to protein synthesis inhibitors such as streptomycin, erythromycin and chloramphenicol. 

### 3.7. 16S rRNA Sequencing and Phylogenetic Tree Results

According to the results obtained above, 11 probiotic candidates (FRY-2, FXHB-6, Fjias-5, FRY-4X, FWJL-4, FZL-2, FRY-6, FSJ-13, FZSJ-7, FXHB-2 and FSJ-4X) were further characterized using the Sanger sequencing method. The sequencing data analysis and the resulting phylogenetic tree demonstrated that these isolates belonged to *L. gasseri*, *L. plantarum* and *L. rhamnosus* strains (Table 4, Figure S1). All isolates’ names, sequence similarity and accession numbers were shown in Table 4. As shown in Table 4, three species of probiotic LAB including *L. gasseri*, *L. plantarum* and *L. rhamnosus* were finally selected as potential probiotics in this study. In addition, the phylogenetic tree was constructed by MEGA7 software (version 7.0) using the bootstrap method (1000 bootstraps have been performed) (Figure S1).

### 3.8. LAB Treatments Inhibit Inflammation in LPS-Treated RAW 264.7 Murine Macrophages 

**Figure 4:**
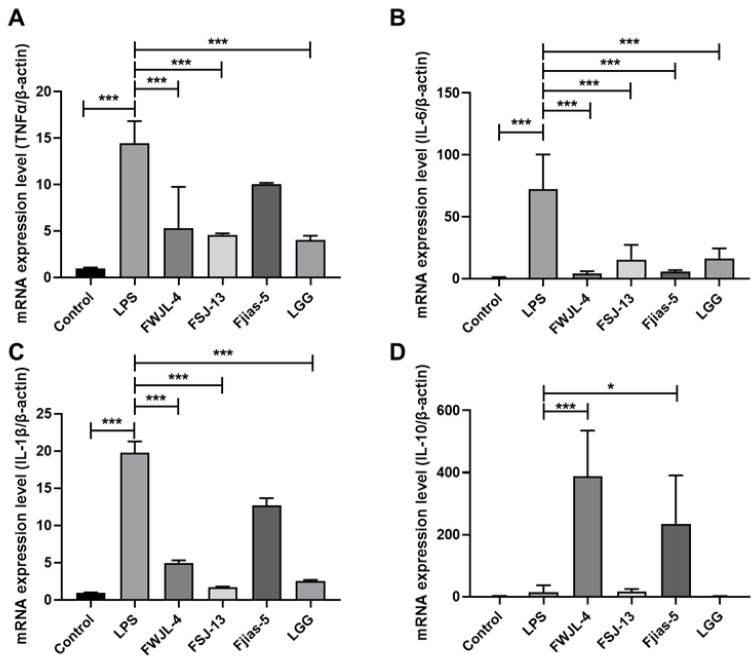
Selected probiotic LAB (*L. gasseri* FWJL-4, *L. plantarum* Fjias-5 and *L. rhamnosus* FSJ-13) show anti-inflammatory effects in LPS-treated RAW 264.7 murine macrophages. (**A**) The mRNA expression level of TNFα.(**B**) The mRNA expression level of IL-6. (**C**) The mRNA expression level of IL-1β. (**D**) The mRNA expression level of IL-10. All data were expressed as mean ± SD (*n* = 3 independent experiments). Statistical significance was defined as * *p* < 0.05; *** *p* < 0.001. Reported statistical significance refers to comparisons with cell-treated with LPS.

Three strains from different species (*L. gasseri* FWJL-4, *L. plantarum* Fjias-5 and *L. rhamnosus* FSJ-13) were selected to evaluate their health-promoting effects. Not surprisingly, LPS treatment significantly enhanced the mRNA expression levels of pro-inflammatory cytokines including TNFα, IL-6 and IL-1β (Figure 4A–C), whereas decreasing IL-10 (Figure 4D), a common anti-inflammatory cytokine. However, cells treated with four strains of probiotic LAB exhibited reduced expression levels of TNFα, IL-6 and IL-1β, and increased expression of IL-10, with a pronounced effect observed for *L. gasseri* FWJL-4 (Figure 4A–D), implying a potential immunomodulatory property of *L. gasseri* FWJL-4. 

### 3.9. LAB Treatments Reduce ROS Production in LPS-Treated RAW 264.7 Murine Macrophages 

**Figure 5:**
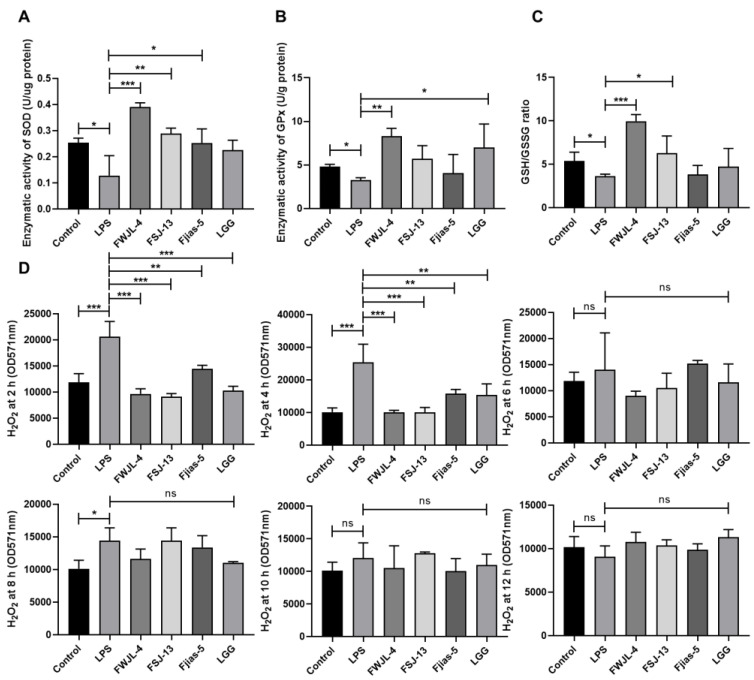
Selected probiotic LAB (*L. gasseri* FWJL-4, *L. plantarum* Fjias-5 and *L. rhamnosus* FSJ-13) enhance antioxidant enzymes and reduce H_2_O_2_ production in LPS-treated RAW 264.7 murine macrophages. (**A**) The enzymatic activities of SOD in different groups. (**B**) The enzymatic activity of GPx in different groups. (**C**) The ratio of GSH/GSSG in different groups. (**D**) The generation of cellular H_2_O_2_ at 2 h, 4 h, 6 h, 8 h, 10 h and 12 h in different groups. All data were expressed as mean ± SD (*n* = 3 independent experiments). Statistical significance was defined as * *p* < 0.05; ** *p* < 0.01; *** *p* < 0.001. “ns”: no significance. Reported statistical significance refers to comparisons with cell-treated with LPS.

Since inflammatory signaling or inflammasome maybe positively regulated by ROS [32,33]. Next, we test whether selected strains can reduce oxidative stress-related features, such as the cellular activity of several antioxidant enzymes and H_2_O_2_ generation. Compare to cells without any treatment, LPS supplementation reduced the activities of antioxidant enzymes while largely increasing the production of H_2_O_2_ (Figure 5A–C). Intriguingly, most LAB treatments significantly enhanced the enzymatic activities of SOD and GPx and the ratio of GSH/GSSG relative to LPS-treated cells (Figure 5A–C). Besides, the generation of cellular H_2_O_2_ was also inhibited by LAB (Figure 5D). In addition, LPS stimulation for 2 h or 4 h significantly increased intracellular H_2_O_2_ generation, which was markedly attenuated by LAB pretreatment (Figure 5D). Besides, only *L. gasseri* FWJL-4 exhibited the highest activity of SOD, GPx and GSH/GSSG ratio relative to reference strain LGG (Figure 5A–C), suggesting the metabolites of *L. gasseri* FWJL-4 might be good antioxidants for reducing oxidative stress.

### 3.10. Selected Probiotic LAB Induce Nuclear Factor-Erythroid 2 Related Factor 2 (Nrf2)-Mediated Signaling Pathways

**Figure 6:**
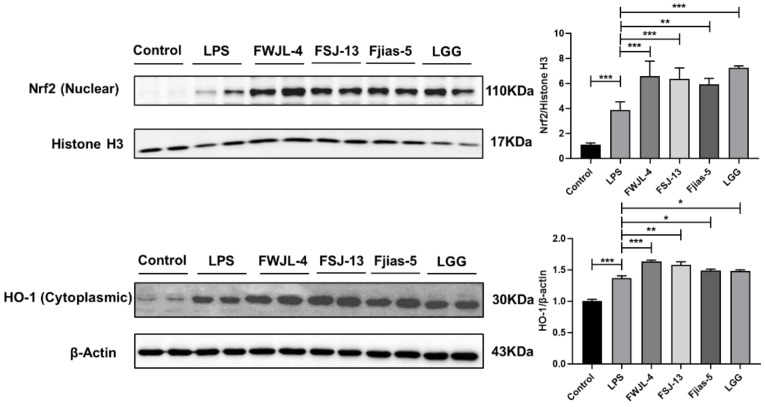
Selected probiotic LAB (*L. gasseri* FWJL-4, *L. plantarum* Fjias-5 and *L. rhamnosus* FSJ-13) induce Nrf2-mediated signaling pathways. All data were expressed as mean ± SD. * *p* < 0.05; ** *p* < 0.01; *** *p* < 0.001 were considered as statistical significance. Reported statistical significance refers to comparisons with cell-treated with LPS.

Since we find the extraordinary anti-inflammatory capacity of selected LAB, we next examine the protein levels of Nrf2, and its downstream antioxidant enzyme heme oxygenase-1 (HO-1). The two proteins are multifunctional modulators of inflammation and oxidative stress-related disorders [34]. In the present study, the expression levels of Nrf2 and HO-1 were increased in LPS-treated RAW 264.7 cells (Figure 6). Consistently, probiotic LAB treatment markedly enhanced the expression levels of Nrf2 and HO-1, with a pronounced effect observed for *L. gasseri* FWJL-4. However, the cells treated with the culture supernatants of *L. plantarum* Fjias-5 exhibited a poorer antioxidant capacity relative to the other strains. Together, the results demonstrate a potential role of metabolites of *L. gasseri* FWJL-4 in reducing cellular oxidative stress.

## 4. Discussion

Over the years, due to multiple health benefits and market demands, probiotics have gained considerable attention. Accumulating studies have been conducted to isolate and characterize potential probiotics from various origins [9,35]. LAB are the most common probiotics found in human GI-tract. This indigenous microbiota plays a major role in maintaining the microbial ecosystem of gut and modulating gut immune homeostasis [36]. 

In this study, we isolated 250 microbial strains from healthy infant feces. The biochemical and morphological characteristics of these strains were then identified using the Gram-staining, the catalase test and analysis of carbohydrate fermentation profiles. 27 Gram-positive and catalase-negative strains were selected. Besides, they could ferment various carbohydrates except for mannitol and sorbitol. As reported, lactose intolerance could cause undesirable gastrointestinal symptoms [37], and the ability of lactose utilization could alleviate lactose intolerance in specific individuals. Furthermore, all strains exhibited abilities to utilize other carbohydrates except mannitol and sorbitol. Thus, they may be related to metabolizing human milk oligosaccharides in infants [38]. 

The safety of bacterial strains is a crucial element in selecting potential probiotics. Firstly, we found that the culture suspensions of selected strains could inhibit the growth of five common pathogens, including both Gram-positive pathogens including *Staphylococcus aureus*, *Listeria monocytogenes* and *Enterococcus faecalis*, and Gram-negative pathogens (*Salmonella enterica* and *Escherichia coli*). These findings indicated that the selected strains with broad-spectrum antimicrobial activities might effective in hospital-acquired infections. Additionally, our strains showed better inhibitory effects than those reported in earlier studies [39,40]. The broad antimicrobial effects of LAB species are attributable to the production of metabolites such as organic acid, antimicrobial peptides, etc. Notably, all selected strains exhibited strong inhibitory effects against *E. faecalis* E27. As reported, due to the exceptional multidrug resistance, most isolates of *E. faecalis* have caused clinical infections that are often hard to treat [41]. However, the antibiotic resistance of *E. faecalis* selected in our study hasn’t been assayed. The inhibitory mechanisms of selected LAB species against pathogens may be associated with inhibition of biofilm formation and exopolysaccharide synthesis [42]. 

Probiotics must be able to survive under harsh conditions including low pH, bile salts and digestive enzymes. In this study, we used artificial saliva and gastric fluid and simulated the digestive tract to evaluate the survival rates of selected strains. The major factor that influences the growth of strains is the acidic circumstance (pH 2). After 2 h of incubation, strains were considered to pass through the stomach. Isolates including FRY-3, Fjias-5, FWJL-4, FRY-6 and FXHB-2 showed a higher survival ability than the reference strain LGG under unfavorable conditions. The results obtained in this study agreed with those from previous studies [43,44,45]. Furthermore, the resistance of probiotics to low pH and bile salts varies greatly among species and strains, suggesting that this survival ability may be strain-specific [23,38]. Resistance to low pH is important for the development of fermented foods such as yogurt and cheese. These foods are often acidic and can affect strain viability. It is reported that the resistance to high bile salts is associated with physiological changes in probiotics such as carbohydrate fermentation and exopolysaccharide production [46]. The composition of membrane proteins and fatty acids and the inhibition of pathogen adhesion to human mucus are also related to the adaption of probiotics to bile salts [47,48]. Therefore, resistance to bile salts is an important trait for strains to compete with pathogens when used in functional foods. 

The ability to colonize the GIT epithelial cells is another important feature for potential probiotic candidates. Here, the strains showing stronger adhesion rates than the reference strain LGG were selected for further tests. Microbial adhesion to epithelial cells is a complex process, which is closely correlated with the physicochemical composition of the probiotic strain cell [1]. The adhesion ability of a probiotic is a crucial trait, as it helps the probiotic compete with pathogenic bacteria to prevent their colonization of the gastrointestinal tract.

The presence of antibiotic resistance genes in probiotics is considered a safety issue as the resistance genes can transfer among the microorganism community of the gut. Our selected strains were all resistant to kanamycin and polymyxin. Consistent with the literature, the resistant ability to kanamycin has been confirmed for most LAB species [23,49]. Besides, strains show resistance to polymyxin, which is a Gram-negative bacteria inhibitor and has no effect on Gram-positive bacteria. Notably, nearly all isolates were resistant to vancomycin, which was in accordance with previous studies [29,50]. However, it is reported that when the antibiotic resistance is intrinsic (chromosomally encoded, non-transferable and non-inducible), the probiotic strains do not constitute a safety concern itself. Thus, the resistant probiotic strains can be used concomitantly or after antibiotic treatment to restore the gut microbiota [51]. In fact, due to the lack of cytochrome-mediated electron transport in *Lactobacillus* genera, and the presence of D-Ala-D-lactate in their peptidoglycan, resistance to streptomycin, kanamycin, and vancomycin is considered to be intrinsic [52]. 

Several antioxidant enzymes including SOD, GPx, GSH, GSSG and HO-1 are secreted to protect cells against oxidative damage. In accordance with other studies, these enzymes were collectively enhanced and ROS production was reduced by our selected LAB [53,54,55]. Nrf2 has long been considered a cytoprotective factor regulating anti-inflammatory and anti-oxidative proteins. In Nrf2-dependent cell antioxidant responses, HO-1 is one of the main effectors, exerting beneficial effects through the protection against cellular oxidative injury, modulation of inflammation and regulation of apoptosis [56,57]. Macrophage polarization is a hallmark of inflammation [58]. As reported, macrophages can be differentiated into two subsets: pro-inflammatory M1 macrophages and anti-inflammatory M2 macrophages. Pro-inflammatory cytokine (IL-1β, TNFα and IL-6) production further causes aggravated inflammation. Besides, excessive ROS production is involved in various chronic inflammation-mediated diseases. Several inflammatory signaling and inflammasome are positively regulated by ROS derived from uncharacterized organelles [59,60]. In oxidative stress and inflammation condition, enhancement of Nrf2/HO-1 expression exerts a crucial role in cell protection [56]. Various oxidative-inducing agents, including LPS, can induce the expression levels of Nrf2 and HO-1 [57]. Our data also showed that LPS increased the levels of Nrf2 and HO-1, however, the selected LAB, especially *L. gasseri* FWJL-4, could further enhance the levels of Nrf2 and HO-1. The high expression levels of Nrf2 and HO-1 can inhibit LPS-activated ROS production, thus decreasing the levels of pro-inflammatory cytokines in RAW 264.7 murine macrophages. These results are consistent with previous reports that probiotics participate in the modulation of inflammation-related diseases by regulating oxidative stress [20,21]. Compare to previous studies, the probiotics screened in the present study exhibited both strong anti-inflammatory and antioxidative properties [61,62,63,64], which were largely attributed to their secondary metabolites. Therefore, herein, we provide a possibility that the metabolites of probiotics maybe functional substances in alleviating inflammation and oxidative stress, which needs to be further explored, however.

## 5. Conclusions

**Figure 7:**
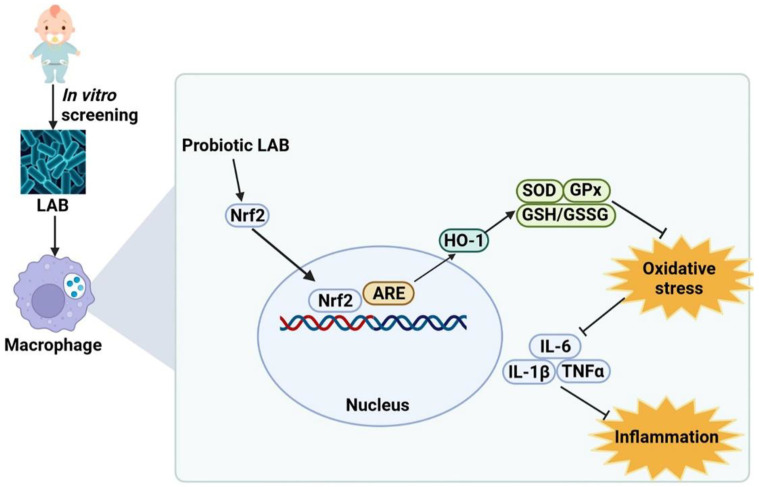
Proposed molecular mechanisms underlying the inhibitory effect of LAB (isolated from infant feces) on the activation of macrophages induced by LPS. LAB activates Nrf2/HO-1 pathway to regulate cellular redox status and reduce oxidative stress, which in turn inhibits inflammation. Nrf2: nuclear factor-erythroid factor 2-related factor 2; ARE: antioxidant responsive element; HO-1: heme oxygenase-1; SOD: superoxide dismutase; GPx: glutathione peroxidase; GSH/GSSG: glutathione/oxidized glutathione; IL-6: Interleukin-6; IL-1β: Interleukin-1β; TNFα: tumor necrosis factor α.

Our study suggests that three selected strains (*L. gasseri* FWJL-4, *L. plantarum* Fjias-5 and *L. rhamnosus* FSJ-13) activate the Nrf2/HO-1 signaling pathway to reduce oxidative stress, thus inhibiting inflammation in macrophages (Figure 7). These strains maybe good candidates in the applications of human trials and may provide a promising therapeutic approach to preventing oxidative stress and inflammation-associated disorders. Our study also provides a theoretical basis for the exploration of probiotic functional metabolites.

## Figures and Tables

**Table 1:** Primers used for RT-qPCR.

Gene		Sequence (5′-3′)	Product Size (bp)	Accession Number
*β-Actin*	Forward	ATGACCCAAGCCGAGAAGG	185	NM_027493
	Reverse	CGGCCAAGTCTTAGAGTTGTTG		
*Tnfα*	Forward	CCACGCTCTTCTGTCTACTG	169	NM_010851.2
	Reverse	ACTTGGTGGTTTGCTACGAC		
*Il-10*	Forward	GGACCAGCTGGACAACATACTGCTA	80	NM_010548.2
	Reverse	CCGATAAGGCTTGGCAACCCAAGT		
*Il-6*	Forward	GAGTCACAGAAGGAGTGGCTAAGG	106	NM_031168.1
	Reverse	CGCACTAGGTTTGCCGAGTAGATCT		
*Il-1β*	Forward	TTGAAAGTCCACCTCCTTACAGA	129	NM_008756
	Reverse	CCGGATAAAAAGAGTACGCTGG		

**Table 2:** **^a^** Results of the antimicrobial activity of selected isolates.

Strains	*Staphylococcus aureus* ATCC 25923	*Salmonella enterica* ATCC 14028	*Escherichia coli* ATCC 25922	*Listeria monocytogenes* ATCC 13932	*Enterococcus faecalis* E27
FRY-2	10.67 ± 0.5	14.3 ± 1	11.67 ± 0.5	12.7 ± 0.5	14.7 ± 1.53
FXHB-6	11.33 ± 0	12.67 ± 1.1	10.3 ± 0.4	13.7 ± 0.5	15.7 ± 0.5
FRY-3	8.7 ± 1.0	8.3 ± 0.5	10.7 ± 0.5	11.3 ± 0.5	15.3 ± 1.1
FMM-1	-	-	-	-	11.3 ± 0.5
FZL-21	11.7 ± 1.1	10.7 ± 0.5	12.3 ± 1.1	11 ± 1.0	17 ± 1.0
Fjias-5	19.3 ± 1.0	14.33 ± 0.5	20.3 ± 0.5	19.67 ± 0.4	19.3 ± 1.1
FWJL-4	14.67 ± 0.5	19.7 ± 1.1	19.33 ± 0.5	14.7 ± 0.5	21.7 ± 0.5
FLY-17	-	-	-	-	10.7 ± 1.1
FZL-2	16.67 ± 1.7	12.7 ± 1.1	15 ± 0	12.67 ± 0.5	15 ± 1.0
FRY-6	15.3 ± 0.5	12.3 ± 1.1	11.67 ± 0.5	14.33 ± 0.5	18.7 ± 0.5
FMM-7	-	-	-	-	-
FSJ-1	12.3 ± 0.5	11.3 ± 0.5	10.6 ± 0.5	9.33 ± 0.5	17.3 ± 1.1
FWJL-5	13.7 ± 0.58	17 ± 1.0	14.7 ± 0.5	13.6 ± 0.5	20.7 ± 1.1
FHHY-7	-	-	-	-	15.3 ± 1.1
FSJ-11	14.33 ± 1.0	15.7 ± 1.1	12.67 ± 0.5	18.3 ± 0.5	18.7 ± 1.5
FSJ-13	18.7 ± 1.5	19.7 ± 0.5	18.3 ± 0.5	17.3 ± 1.1	20.7 ± 1.1
FZSJ-7	11.3 ± 0.4	12.7 ± 0.5	14.33 ± 0.5	12.7 ± 0.5	18 ± 1.0
FSJ-6	-	-	-	8.7 ± 1.1	-
FXHB-2	12.67 ± 0.5	17 ± 1.0	19 ± 1.0	17.3 ± 1.15	18.67 ± 0.5
FSJ-4X	14.33 ± 0.5	17 ± 0.5	13.3 ± 0.5	17 ± 1.0	19 ± 1.0
FLT6-11	8.67 ± 0.4	-	-	8.3 ± 0.5	-
FXHB-2X	9.3 ± 1.1	8.3 ± 0.5	16 ± 1.0	17.3 ± 1.1	15.3 ± 0.5
FRY-4X	9.7 ± 0.5	14.3 ± 1.15	13.7 ± 0.5	11 ± 1.0	20.7 ± 1.1
FSJ-2	10.3 ± 0.4	9.7 ± 0.5	9 ± 1.0	8.7 ± 1.53	15.3 ± 0.5
FHHY-1	16.3 ± 0.5	15 ± 1.0	12.7 ± 0.5	15.7 ± 0.5	20.67 ± 1.15
FZYY-3	10.7 ± 0.5	9.67 ± 0.5	8.33 ± 0.5	10.7 ± 0.5	15.67 ± 0.5
FQM-3X	-	-	-	-	-
Penicillin	32.7 ± 0.5	30.3 ± 1.15	35.7 ± 0.5	33.3 ± 0.4	38.67 ± 0

^a^ No inhibition (–).

**Table 3:** Antibiotic resistance of tested strains.

Strains	Name	^a^ MIC (μg/mL)							
		K	P	V	E	A	S	C	PO
FRY-2	*Lactobacillus gasseri*	16	4	4	<2	<2	2	2	≥1024
FXHB-6	*Lactobacillus gasseri*	16	32	2	<2	<2	2	2	≥1024
FRY-3	*Lactobacillus gasseri*	16	4	2	<2	2	2	2	1024
FZL-21	*Lactobacillus gasseri*	64	4	2	<2	2	2	2	≥1024
Fjias-5	*Lactiplantibacillus plantarum*	512	<2	2	<2	<2	16	8	≥1024
FRY-4X	*Lactobacillus gasseri*	<2	8	2	<2	<2	2	<2	≥1024
FWJL-4	*Lactobacillus gasseri*	64	8	2	<2	8	16	2	1024
FZL-2	*Lactobacillus gasseri*	64	16	4	<2	64	2	2	≥1024
FRY-6	*Lactobacillus gasseri*	64	16	8	8	4	8	2	≥1024
FSJ-1	*Lacticaseibacillus rhamnosus*	512	512	≥1024	8	16	32	2	≥1024
FWJL-5	*Lactobacillus gasseri*	64	32	2	<2	8	2	2	1024
FSJ-11	*Lacticaseibacillus rhamnosus*	512	2	≥1024	2	<2	32	<2	≥1024
FSJ-13	*Lacticaseibacillus rhamnosus*	≥1024	<2	≥1024	2	2	128	<2	≥1024
FZSJ-7	*Lactobacillus gasseri*	16	4	≥1024	<2	<2	<2	<2	≥1024
FXHB-2	*Lactobacillus gasseri*	16	4	≥1024	<2	<2	<2	<2	≥1024
FSJ-4X	*Lacticaseibacillus rhamnosus*	32	<2	≥1024	2	<2	32	<2	≥1024
FXHB-2X	*Lactobacillus gasseri*	128	4	≥1024	<2	8	4	<2	≥1024
LGG	*Lacticaseibacillus rhamnosus*	256	8	≥1024	<2	32	64	8	≥1024

^a^ K: kanamycin, P: penicillin, V: vancomycin, E: erythromycin, A: ampicillin, S: streptomycin, C: chloramphenicol, PO: polymyxin.

**Table 4:** 16S rRNA sequencing of candidate probiotics based on the percentage similarity of the sequence found in the GenBank database.

Names	Sequencing Results	Identity	Accession Number
FRY-2	*Lactobacillus gasseri*	97.90%	MZ220367
FXHB-6	*Lactobacillus gasseri*	99.79%	MZ220368
Fjias-5	*Lactiplantibacillus plantarum*	99.93%	MZ220369
FRY-4X	*Lactobacillus gasseri*	99.79%	MZ220370
FWJL-4	*Lactobacillus gasseri*	99.79%	MZ220371
FZL-2	*Lactobacillus gasseri*	99.79%	MZ220372
FRY-6	*Lactobacillus gasseri*	93.73%	MZ220373
FSJ-13	*Lacticaseibacillus rhamnosus*	99.80%	MZ220374
FZSJ-7	*Lactobacillus gasseri*	99.79%	MZ220375
FXHB-2	*Lactobacillus gasseri*	99.86%	MZ220376
FWJL-5	*Lactobacillus gasseri*	99.79%	MZ220377
FSJ-4X	*Lacticaseibacillus rhamnosus*	99.86%	MZ220378

## Data Availability

The data is contained within this article.

## Supplementary Materials

The following supporting information can be downloaded at: https://www.mdpi.com/article/10.3390/antiox11071246/s1, Figure S1: The phylogenetic tree shows the relationships between different LAB strains.
