# Supplementary material for: Novel Probiotic Lactic Acid Bacteria Were Identified from Healthy Infant Feces and Exhibited Anti-Inflammatory Capacities
Source: Antioxidants (Basel). 2022 Jun 24;11(7):1246. doi: 10.3390/antiox11071246 (PMC9312049; doi:10.3390/antiox11071246)
Supplement: Supplementary file 1 [file antioxidants-1743538-supplementary.pdf › antioxidants-1743538-supplementary.pdf]

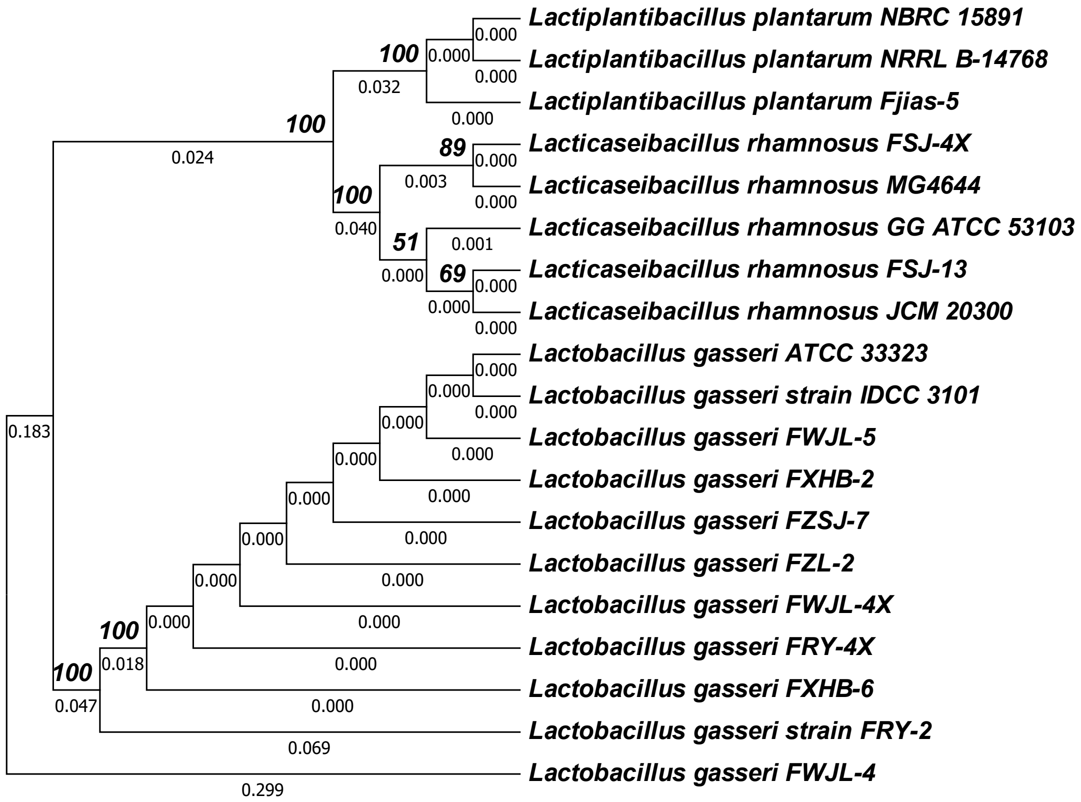

Figure S1. The phylogenetic tree shows the relationships between different LAB strains. Numbers at nodes are bootstrap values. 1000 bootstraps were performed. Nucleotide sequence data used for analysis are available in the Genbank database under the accession numbers: MZ220367 (FRY-2), MZ220368 (FXHB-6), MZ220369 (Fjias-5), MZ220370 (FRY-4X), MZ220371 (FWJL-4), MZ220372 (FZL-2), MZ220373 (FRY-6), MZ220374 (FSJ-13), MZ220375 (FZSJ-7), MZ220376 (FXHB-2), MZ220377 (FWJL-5), MZ220378 (FSJ-4X).
